# STAT1 overexpression triggers aplastic anemia: a pilot study unravelling novel pathogenetic insights in bone marrow failure

**DOI:** 10.1007/s10238-023-01017-0

**Published:** 2023-02-24

**Authors:** Antonio Giovanni Solimando, Vanessa Desantis, Carmen Palumbo, Carolina Marasco, Fabrizio Pappagallo, Monica Montagnani, Giuseppe Ingravallo, Sebastiano Cicco, Rosa Di Paola, Paula Tabares, Andreas Beilhack, Franco Dammacco, Roberto Ria, Angelo Vacca

**Affiliations:** 1 Unit of Internal Medicine “Guido Baccelli”, Department of Precision and Regenerative Medicine and Ionian Area (DiMePRe-J), University of Bari “Aldo Moro” Medical School, Bari, Italy; 2 Section of Pharmacology, Department of Precision and Regenerative Medicine and Ionian Area (DiMePRe-J), University of Bari “Aldo Moro” Medical School, Bari, Italy; 3 Section of Pathology, Department of Precision and Regenerative Medicine and Ionian Area (DiMePRe-J), University of Bari “Aldo Moro” Medical School, Bari, Italy; 4 Research Unit of Diabetes and Endocrine Diseases, Fondazione IRCCS Casa Sollievo Della Sofferenza, Viale Cappuccini, 71013 San Giovanni Rotondo, Foggia Italy; 5 Department of Medicine II, University Hospital of Würzburg, Würzburg, Germany; 6 Interdisciplinary Center for Clinical Research Laboratory, University Hospital of Würzburg, Würzburg, Germany

**Keywords:** Bone marrow failure, Aplastic anemia, STAT1, Gain-of-function, Inborn error of immunity, Immunodeficiency

## Abstract

We identified STAT1 gain of function (GOF) in a 32-year-old female with pallor, weakness, cough, and dyspnea admitted to our Division of Medicine. She had severe oral ulcers (OU), type 1 diabetes (T1DM), and pancytopenia. Bone marrow (BM) biopsy showed the absence of erythroid precursors. Peripheral blood parameters such as neutrophils < 500/mL, reticulocytes < 2%, and BM hypo-cellularity allowed to diagnose severe aplastic anemia. A heterozygous variant (p.520T>C, p.Cys174Arg) of STAT1 was uncovered. Thus, p.Cys174Arg mutation was investigated as potentially responsible for the patient's inborn immunity error and aplastic anemia. Although STAT1 GOF is rare, aplastic anemia is a more common condition; therefore, we explored STAT1 functional role in the pathobiology of BM failure. Interestingly, in a cohort of six patients with idiopathic aplastic anemia, enhanced phospho-STAT1 levels were observed on BM immunostaining. Next, the most remarkable features associated with STAT1 signaling dysregulation were examined: in both pure red cell aplasia and aplastic anemia, CD8^+^ T cell genetic variants and mutations display enhanced signaling activities related to the JAK-STAT pathway. Inborn errors of immunity may represent a paradigmatic condition to unravel crucial pathobiological mechanisms shared by common pathological conditions. Findings from our case-based approach and the phenotype correspondence to idiopathic aplastic anemia cases prompt further statistically powered prospective studies aiming to elucidate the exact role and theragnostic window for JAK/STAT targeting in this clinical context. Nonetheless, we demonstrate how a comprehensive study of patients with primary immunodeficiencies can lead to pathophysiologic insights and potential therapeutic approaches within a broader spectrum of aplastic anemia cases.

**Supplementary Information:**

The online version contains supplementary material available at 10.1007/s10238-023-01017-0.

## Introduction

Pure red cell aplasia (PRCA) and aplastic anemia are deadly illnesses resulting from bone marrow (BM) failure. Patients usually complain of severe anemia, weakness, infections, and bleeding. Unleashed T cell-mediated autoimmune mechanisms are often involved [1]. Nonetheless, inborn errors of immunity, genetic diseases such as Fanconi anemia, dyskeratosis congenita and telomere disease, as well as unintentional exposure to physical and chemical toxins, should also be considered in the differential diagnosis [1]. Findings supporting an immune-mediated mechanism of PRCA and aplastic anemia involve, but are not limited to, the presence of BM T cells, enhanced number of stem-like memory CD8^+^ T cells in the peripheral blood (PB), and the bona fide effectiveness of immunosuppressive therapy [1, 2].

The underlying pathophysiology driving chronic mucocutaneous candidiasis (CMC) in STAT1 gain-of-function (GOF) has been correlated to enhanced Th1 activity and decreased Th17 function. Moreover, STAT1 GOF individuals are also characterized by several Th1 polarized cells higher than Th17 [3]. Based on this observation, we wondered whether the immune system could play a role in inborn errors of immunity characterized by a multisystem immune dysregulation and by BM failure.

To this aim, we describe a case-based approach to STAT1 GOF variant while providing new insights to help clinicians optimize molecular testing for patients with complex primary immunodeficiencies. Concomitantly, our promising results of a precision genomic immunology approach, support an improved strategy that might potentially be extended to a less rare clinical condition such as idiopathic aplastic anemia.

## Case-based study of aplastic anemia

**Fig. 1:**
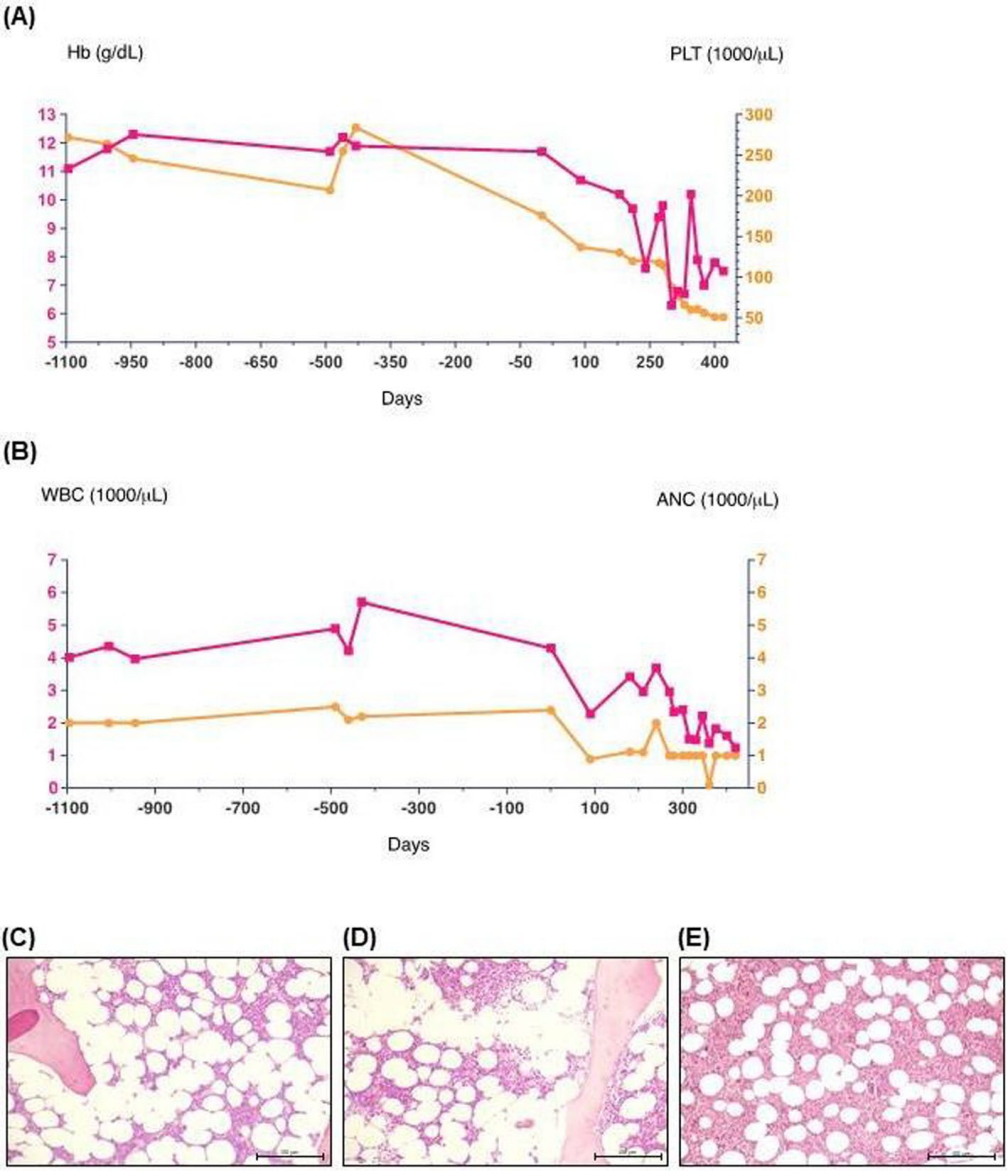
Case-based approach to aplastic anemia. **A** Timeline (days) of peripheral blood (PB) laboratory values, identified pancytopenia with a hemoglobin (Hb, purple) level of 7.6 g/dL (reference range 12–16 g/dL) without signs of hemolysis and a platelet (PLT, yellow) count of 51,000/µL (reference range 140,000–430,000 PLTs/µL). **B** Timeline (days) of a white blood cell (WBC, purple) count showed values of 1,240 cells/µL (reference range 4,500–11,000 cells/µL) and an absolute neutrophil (ANC, yellow) count of 1,000 cells/µL (count nadir reported, reference range 1,800–7,700 cells/µL). **C**–**E** Representative images of immunohistochemical staining in formalin-fixed 4 µm BM sections from the iliac crest. **C** Stat1 GOF variant patient, **D** idiopathic aplastic anemia and **E** non-aplastic benign anemia were stained with Haematoxilin-Eosin (DAKO, Golstrup, Denmark). Representative images showed a ratio of hematopoietic marrow to adipose tissue of 1:3 in our patient with STAT1 GOF variant (hypocellular condition to the patient's age). The erythroid and myeloid series markedly decreased, and a prevalence of more mature precursors was revealed. Sections were examined using an Olympus microscope (Olympus Italia, Rozzano, Italy). Original magnification × 10; scale bar = 100 µm

A 32-year-old female was admitted with symptoms of pallor, weakness, cough, and dyspnea of 3-weeks’ duration. On examination, she was pale but well-appearing with a body mass index (BMI) of 19,5 kg/m^2^. She had painful oral ulcers, a history of oral CMC on the soft palate, recurrent pneumonia and type 1 diabetes mellitus (T1DM). Her laboratory results were remarkable for pancytopenia with a hemoglobin (Hb) level of 7,6 g/dL (reference values 12–16 g/dL), white blood cell (WBC) = count nadir of 1,240 cells/µL (reference values 4,500–11,000 cells/µL), an absolute neutrophil cell (ANC) count of 1,000 cells/µL (reference range 1,800–7,700 cells/µL), and a platelet (PLT) count of 51,000/µL (reference range 140,000–430,000 PLTs/µL) (Figure 1A, B). Autoimmune and other hemolyses were ruled out. The workup elucidating the laboratory values as part of an infectious and rheumatologic workup of pancytopenia has also been performed (Supplementary table 1). She was transfused with red blood cells, and a BM biopsy showed a ratio of hematopoietic marrow to adipose tissue of 1:3 (hypocellular to the patient's age) (Figure 1C–E). The erythroid series was markedly decreased and showed a prevalence of more mature precursors. The myeloid series was also reduced, with a prevalence of more mature precursors. The myeloid to erythroid ratio was 5:1. The lymphoid series was represented by some cell aggregates of reactive significance, of small size and immunophenotype T. Megakaryocytes were numerically within normal limits but of variable size. These findings allowed us to exclude myelopathies or BM fibrosis secondary to metastatic cancer cells. Gastroscopy and colonoscopy, as well as gynecological examination, were also unremarkable. When the patient was previously studied at other medical centers, alterations of the Autoimmune regulator (AIRE) gene supporting a possible diagnosis of Autoimmune Polyendocrinopathy-Candidiasis-Ectodermal-Distrophy (APECED), and a deficit of phagocyte bactericidal function assessed by nitro-blue tetrazolium test was ruled out. Laboratory testing was negative for Fanconi anemia, telomere disease and Marchiafava-Micheli disease. In a multiparametric flow cytometry analysis of BM, B CD19+CD20+ lymphocytes were absent, and a frequency of 98% CD3+ T cells among all lymphocytes with a ratio CD4:CD8 of 0.5 was observed. Although the patient’s PLT count remained relatively preserved, the occurrence of PB neutrophils < 500/mL, reticulocytes < 2% and BM hypo-cellularity confirmed the diagnosis of severe aplastic anemia. Since childhood, the patient suffered from recurrent severe oral ulcers, treated on an as-needed basis with prednisone 10 mg/daily. She also suffered from T1DM, opportunistic infections and recurrent pneumonia and CMC. CMC episodes causing her main discomfort were treated with nystatin oral suspension. The patient and her family were of Caucasian descent. Her family history was remarkable that her mother also suffered from T1DM since early childhood. Her grandmother was diagnosed with autoimmune thyroiditis. The construction of a pedigree chart suggested an autosomal-dominant inheritance pattern for the T1DM phenotype with severe oral and esophageal candidiasis.

## Identification of a pathogenic STAT1 p.520T>C, p.Cys174ARG variant

**Fig. 2:**
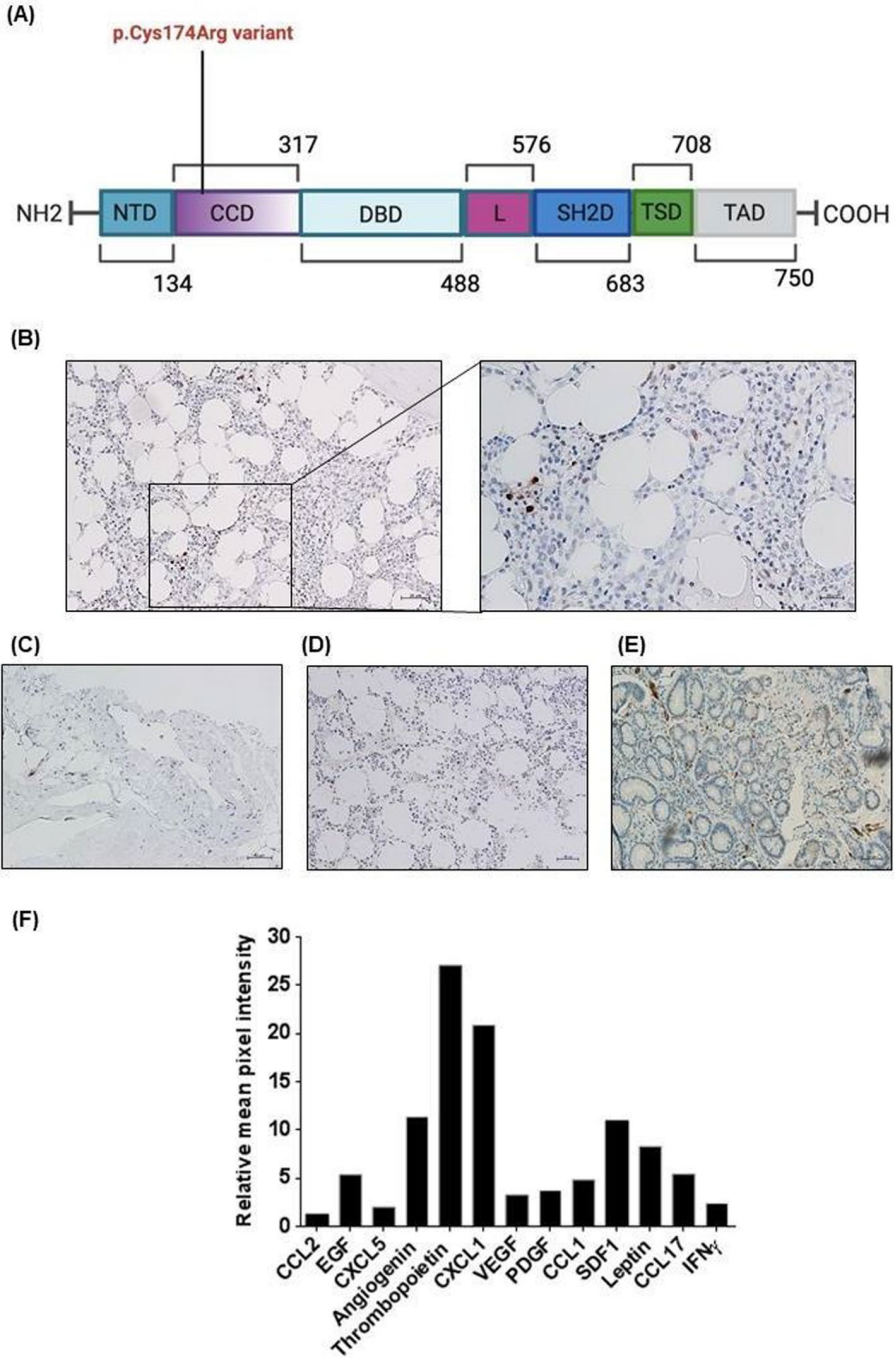
STAT1 expression in aplastic anemia cases. **A** Reported gain-of-function (GOF) mutations of STAT1. GOF mutations are shown in red. Specifically, the p.Cys174Arg variant has been identified in our patient and classified as pathogenic (see text for details). A heterozygous variant (p.520T>C, p.Cys174Arg) in the coiled-coil domain of the STAT1 gene, showing in vitro gain of function (GOF) activity, identified in our patient and was confirmed by Sanger sequencing. **B–E** Immunohistochemistry staining for p-STAT1 on formalin-fixed 4 µm bone marrow sections from the iliac crest of (**B**) our patient with STAT1 GOF variant (a magnified box is also provided), **C**, of a representative patient with idiopathic aplastic anemia, **D** of a representative patient with benign anemia and **E** of gastric positive section used as positive control. Sections were examined using an Olympus microscope (Olympus Italia, Rozzano, Italy). Original magnification ×20 and × 40; scale bar = 50 µm/20 µm. Note the increased p-STAT1 expression in STAT1 GOF variant and in idiopathic aplastic anemia compared to benign anemia cases (representative figures are provided). **F** Human Cytokine Antibody Array. Cytokine levels are quantified with Kodak Molecular Imaging Software version 2.0.0 (Eastman Kodak Co., Rochester, NY)

Considering the complexity of the clinical scenario, characterized by T1DM, recurrent infections, aphthous lesions and the unclear etiology of her aplastic anemia, a whole-exome sequencing of genomic DNA from the patient was performed. Briefly, genomic DNA was extracted from whole blood by means of QIAmp DNA Blood Kit (Qiagen Inc., Valencia, CA, USA), according to manufacturer’s instructions. Libraries were obtained using SureSelectXT-CCP17 (Agilent Technologies, Santa Clara, California), sequenced on NextSeq-500 (Illumina Inc) and then generated reads aligned to the human genome reference sequence (assembly GRCh37/hg19). At a reading depth greater than 30, regions of interest were covered > 98%. Sequencher-v5.4.6 Software (GeneCodes, Ann Arbor, MI, USA) carried out references-based assembly, and Alamut Visual version 2.10 Interactive Biosoftware analyzed and interpreted variations on a genomic level (SOFHiAGENETICSTM, Saint Sulpice, Switzerland). A heterozygous variant (p.520T>C, p.Cys174Arg, rs387906763) of STAT1 was identified (Figure 2A), which, according to the ACMG/AMP guidelines, was likely pathogenic. It is, indeed, a missense variant located in a mutational hotspot region in a gene with low rate of benign missense variation and in which missense mutations are a common mechanism of disease. Finally, it is absent in gnomAD population database. Moreover, its frequency in the general population interrogating the public databases (i.e., dbSNP, gnomAD) is unknown. We could confirm our results in a double blinded fashion.

In addition, case-based studies have prompted specific standards for determining the causality of genotypes for given phenotype [5]; it has been described in significant family members with CMC/recurrent infections/T1DM and it has been shown to have GOF activity [4]. Thus, the p.Cys174Arg mutation was considered causative of the patient’s inborn error of immunity. We excluded the most common immune-mediated viral and bacterial causes. Since STAT1 GOF is associated with aplastic anemia [5], our patient's p.Cys174Arg variant was deemed causal for her aplastic anemia as well. However, the possibility that aplastic anemia developed coincidentally with the patient's STAT1 GOF mutation cannot be ruled out.

## Validations of STAT1 overexpression in idiopathic aplastic anemia case series

Remarkably, immunohistochemistry (IHC) performed on the BM specimens confirmed overexpression of phospho-STAT1 (p-STAT1), thus corroborating genomic data (Fig. 2B). This prompted us to wonder whether aplastic anemia without a congenital STAT1 GOF variant might also have a similar pattern of STAT1 hyperactivation. Four out of 6 idiopathic aplastic anemia cases, compared to 6 cases of non-aplastic anemia, displayed increased STAT1 activity, showing p-STAT1 staining of BM core sections (gastric sections have been used as positive controls according to monoclonal antibody manufacturer’s indications) (Fig. 2C–E). In more details, p-STAT1 expression was examined by IHC on BM sections from 6 cases of idiopathic aplastic anemia and 6 of non-aplastic benign anemia. Each section of 4 μm thickness was cut from formalin-fixed and paraffin-embedded histological material and stained with an indirect immunoperoxidase method using the BenchMark XT automated staining instrument (Ventana Medical Systems, Tucson, AZ, USA). A p-STAT1 rabbit monoclonal antibody (58D6 clone, Cell Signaling) was used at 1:600 dilution.

When cytokines levels were measured in sera obtained from BM aspirate of our patient with STAT1 GOF mutation (Human Cytokine Antibody Array, Abcam) (Figure 2F), expression of angiogenin, CXCL1, CCL1, CCL2, CCL17, EGF, leptin, thrombopoietin, SDF1 and interferon γ (IFN­γ), was deemed significantly higher than in negative control. These data are consistent with previous findings regarding the cytokine profile characteristic of aplastic anemia [6].

## Discussion

The Janus kinase (JAK) pathway regulates hematopoietic cascade, immune processes, via a complex signaling cascade involving STAT1 as a downstream transcription factor [7]. Autosomal-dominant clinical conditions characterized by GOF variants of STAT1 have been reported in patients with increased viral and opportunistic infections and immune dysregulation phenotype [8, 9].

**Fig. 3:**
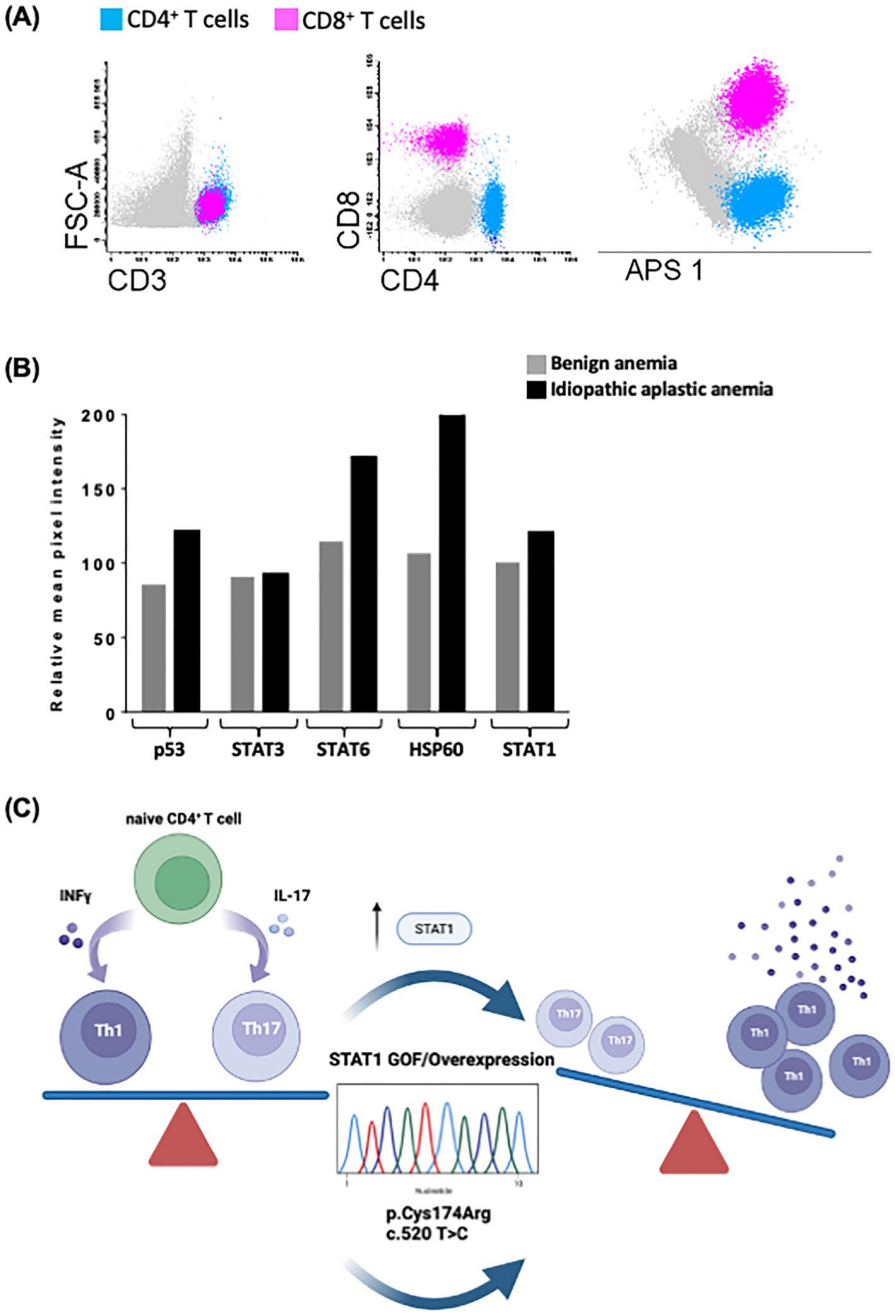
**A** Multiparametric flow cytometry analysis of bone marrow cells. Automatic population separator (APS) plot provided the best cell cluster separation, to confirm the identity of CD4+ and CD8+ T cells. Grey color: other nucleated populations.** B** Human Phospho-kinase Array. Levels of phosphorylated proteins were quantified with Kodak Molecular Imaging Software version 2.0.0 (Eastman Kodak Co., Rochester, NY). **A** and **B** representative cases of 6 independent experiments from idiopathic aplastic anemia and benign anemia cases.** C** The finding of the heterozygous variant (p.520T>C, p.Cys174Arg) in the coiled-coil domain of STAT1 gene, showing gain of function (GOF) activity paved the way for idiopathic aplastic anemia cases investigation. A boosted STAT1 pathway activation unleashes a Th1 phenotype differentiation, driving a variable clinical phenotype, including bone marrow failure. Created with BioRender.com, Publication license n. YK250GFR64

The pathophysiological mechanisms driving an increased predisposition to CMC in STAT1 GOF have been correlated to an altered balance between Th1 and Th17 cells [10, 11]. Indeed, individuals carrying STAT1 GOF harbor enhanced levels of Th1 over Th17 cells [3, 4], as confirmed by the immunophenotyping performed within our case series (Figure 3A–C). A boosted STAT1 pathway activation unleashes a Th1 phenotype differentiation, mainly characterized by IFN­γ production. A loss in the homeostasis of STAT3-mediated Th17 polarization and a lack of IL-17A and Th17 secreted cytokines parallel the Th1 overactivation and lead to an imbalanced defense against fungal infections [3, 4, 12].

Type-I IFN signaling is crucial for host defense and clearance of viral infections. The canonical type I IFN signaling includes activation and dimerization of STAT1 and STAT2, which form a complex with IRF9 to induce the expression of antiviral response genes [13]. STAT1 is critical in this pathway as both STAT1 knockout animal models and patients with loss of function mutations fail to clear viral infections [14]. Unexpectedly, patients with STAT1 GOF also develop chronic infections. Since natural killer (NK) cells are pivotal for viral clearance, a STAT1 GOF can alter NK cell function during viral infection. Indeed, NK cells respond to type I IFN [15]. At steady-state, STAT4 levels are much higher than STAT1 and upon exposure to type I IFN STAT4 is in charge of the majority of changes in gene expression [16]. Later during viral infections STAT1 protein level increases and takes the lead role in IFN signaling.

Thus, STAT1 GOF can alter IFN signaling in NK cells, contributing to their dysfunction during viral infection. In a mouse model with STAT1 GOF, NK cells exhibit aberrant STAT1 activation after IFN-alpha exposure, while STAT4 activation remains unchanged [16]. Thus, the balance between STAT1 and STAT4 activation in NK cells would be altered during viral infections. Finally, in an elegant animal model, it was shown that CD4^+^ and CD8^+^ T cells were characterized by enhanced IFN-γ production as an underlying driver of STAT1-related loss of the mucosal barrier [17]. Consequently, those animals display a higher predisposition to CMC [17]. Type 1 cytokines seem to play a central role, mainly orchestrated by IFN-γ, unleashing Fas overactivation on CD34 cells and driving apoptosis [18]. In light of these findings, it is worthwhile to consider whether JAK inhibitor therapy may be beneficial for aplastic anemia patients with STAT1 hyperactivation [19, 20]. This question should be addressed in statistically powered studies.

BM failure syndromes include aplastic anemia, PRCA, myelodysplastic syndrome, and BM fibrosis induced by metastatic cancer. Detecting hypoproliferative anemia is a clinical challenge, profoundly impacting the patients' quality of life and often affecting their prognosis [1, 18]. Inborn errors of immunity are no exception, being also characterized by enhanced risk of cardinal phenotype ancillary to anemia, namely bilinear cytopenia or pancytopenia, as well as infections and inflammatory syndromes [19]. Interestingly, in two studies just published while our investigation was undergoing, others uncovered STAT1 GOF to be a predictive marker for sensitivity to JAK1/STAT1 inhibition [20, 21]. Nonetheless, to our knowledge, the present research represents the first report of the p.Cys174Arg STAT1 GOF variant in a patient with an inborn error of immunity developing aplastic anemia. A case-based study of patients with rare primary immunodeficiency demonstrates how careful investigations may provide pathophysiologic insights and suggest potential therapies for aplastic anemia and inborn immune errors that may be more broadly applicable [22, 23].

## Conclusions

Despite rare, aplastic anemia is more common than STAT1 GOF. We identified this inborn error of immunity phenotype with our patient’s STAT1 GOF mutation in mind. We found that 4 out of 6 cases in a cohort of idiopathic aplastic anemia also uncovered enhanced pSTAT1 levels on BM immunostaining. Thus, the STAT1 signaling dysregulation has remarkable features: in PRCA and aplastic anemia, CD8^+^ T cell genetic variants and mutations are enriched for signaling related to the JAK-STAT pathway [24, 25]. The data from our case and the phenotype correspondence to idiopathic aplastic anemia cases prompt further studies aiming to elucidate the exact role and theragnostic window for JAK/STAT targeting in this clinical context. Inborn errors of immunity can therefore represent a paradigmatic condition to unravel crucial pathobiological mechanisms shared by common pathological conditions.

### Supplementary Information

Below is the link to the electronic supplementary material.

Supplementary file1 (DOCX 16 KB)
