# Supplementary material for: STAT1 overexpression triggers aplastic anemia: a pilot study unravelling novel pathogenetic insights in bone marrow failure
Source: Clin Exp Med. 2023 Feb 24;23(6):2687–94. doi: 10.1007/s10238-023-01017-0 (PMC10543574; doi:10.1007/s10238-023-01017-0)
Supplement: Supplementary file 1 — Supplementary file1 (DOCX 16 KB) [file 10238_2023_1017_MOESM1_ESM.docx]

## Reference Range Presentation Value(T0) Nadir Value +4months(T1)

Hemoglobin (g/dl) 12.0–16.0 7.6 6.3 6.7

Mean corpuscular volume (fL) 80-94 103 103 84.8

Hematocrit (%) 36.0–46.0 25.2 25.2 17.9

Reticulocytes (%) 0.5-2.5 2.2 0.5 0.5

White-cell count (per µl) 4500–11,000 3.77 1.48 1.48

Differential count (%)

Neutrophils 40–70 62.1 51.1 51.1

Lymphocytes 22–44 28.1 41.4

Monocytes 4–11 9.5 7.3

Eosinophils 0–8 0.2 0

Platelet count (per µl) 150,000–400,000 138,000 66,000 66,000

## Chemistries

Sodium (mmol/liter) 135–145 141

Potassium (mmol/liter) 3.4–5.0 3.7

Chloride (mmol/liter) 98–108 107

Carbon dioxide (mmol/liter) 23–32 29

Urea nitrogen (mg/dl) 8–25 27

Creatinine (mg/dl) 0.60–1.50 0.54

Glucose (mg/dl) 70–110 128

Iron Saturation (%) 14-50 96%

Total iron binding capacity (ug/dL) 230-404 230

Ferritin (ug/L) 20-300 651

Folate (ng/mL) >4.7 21.3

Vitamin B12 (pg/mL) >231 64

Lactate dehydrogenase (IU/liter) 110–210 157

Alanine aminotransferase (U/liter) 10–55 39

Aspartate aminotransferase (U/liter) 10–40 40

Bilirubin (mg/dl)

Total 0.0–1.0 0.8

Direct 0.0–0.4 <0.2

## Infectious Workup

Human Immunodeficiency Virus negative

Parvovirus IgM negative IgG positive, PCR negative

Hepatitis B Virus Immune

Hepatitis C Virus antibody negative

Cytomegalovirus IgM negative, IgG positive PCR 2935 copies PCR negative

Epstein Barr Virus Viral Capsid Antigen IgM negative, IgG positive, PCR negative

## Rheumatologic Workup

Antinuclear Antibody negative

anti-dsDNA antibody negative

anti-Smith antibody negative

anti-Ro antibody negative

anti-La antibody negative

anti-ribonucleoprotein antibody negative

anti-neutrophil cytoplasmic antibody negative

anti-cyclic citrullinated protein antibody negative

Rheumatoid factor negative

Celiac autoantibody panel negative
